# Supplementary material for: Cognitive Functions, Theory of Mind Abilities, and Personality Dispositions as Potential Predictors of the Detection of Reciprocity in Deceptive and Cooperative Contexts through Different Age Groups
Source: Behav Sci (Basel). 2023 Dec 10;13(12):1007. doi: 10.3390/bs13121007 (PMC10741168; doi:10.3390/bs13121007)
Supplement: Supplementary file 1 [file behavsci-13-01007-s001.zip › behavsci-2721337-supplementary.pdf]

Supplementary materials

Cognitive functions, Theory of Mind Abilities, and Personality Dispositions as Potential Predictors of the Detection of Reciprocity in Deceptive and Cooperative Contexts through Different Age-Groups

1. *AGE DIFFERENCES IN COGNITIVE MEASURES*

Descriptive statistics are presented in table S1 split by age groups. The results of the one-way ANOVAs and post hoc analyses indicated age-related differences in fluid abilities ($F_{(2,304)}$= 67.56, p<.001) and working memory ($F_{(2,304)}$=30.39, p<.001) between all age groups. Younger adults performed better in both cognitive measures compared to middle aged and older adults and middle-aged adults demonstrated greater cognitive abilities than older adults.

Table S1 : Descriptive statistics for the cognitive measures by age group

	Younger adults (N = 98)		Middle-aged adults (N=106)		Older adults (N=103)	
	Mean (SD)	range	Mean (SD)	Range	Mean (SD)	Range
Fluid abilities	42.87 (3.72)	27-49	39.25 $^{(***)}$ (5.17)	14-47	32.29 $^{(***, +++)}$ (9.37)	5-47
Working memory	5.37 (1.33)	2-8	4.77 $^{(**)}$ (1.25)	2-8	3.91 $^{(***, +++)}$ (1.42)	1-8

Note: FI=Fluid intelligenceA significant difference from the young adult group is represented by (***) p<.001, (**) p<.01, (*) p<.05; A significant difference from the middle-aged group is represented by (+++) p<.001, (++) p<.01, (+) p<.05.

2. *AGE DIFFERENCES IN THE DIFFERENT PERSONALITY DISPOSITIONS*

The descriptive statistics for all the personality dispositions can be found in table S2.

No significant age differences were found in the overall agreeableness scale ($F_{(2,304)}$=.69, p=.50), but there was a marginal age difference found for cordiality ($F_{(2,304)}$=2.36, p=.096) but not cooperativeness ($F_{(2,304)}$=1.65, p=.193) when agreeableness was

subdivided into its two dimensions. Older adults scored marginally higher on the cordiality dimension scale than younger adults. However, old and middle-aged adults demonstrated a significantly greater altruistic attitude ($F_{(2,304)}$=12.52, p<.001) and tendency towards empathic concern ($F_{(2,304)}$=7.45, p<.001) than younger adults.

Table S2 : Descriptive statistics for the personality dispositions divided by age group

	Younger adults (N = 98)		Middle-aged adults (N=106)		Older adults (N=103)	
	Mean (SD)	Range	Mean (SD)	Range	Mean (SD)	Range
BFQ agreeableness	79.12 (11.02)	47-101	80.80 (10.04)	46-108	80.39 (10.67)	56-102
cordiality	36.73$^{(.)}$ (6.83)	22-50	37.71 (6.00)	23-54	38.69 (6.30)	23-54
cooperativeness	42.39 (5.42)	24-51	43.09 (5.56)	23-54	41.7 (5.65)	28-54
ECRC altruistic scale	17.79 $^{(***,+++)}$ (3.19)	10-25	19.58 (3.13)	12-25	19.87 (3.25)	10-25
IRI empathic concern	26.1$^{(***,+)}$ (4.05)	16-35	27.52 (4.46)	16-35	28.38 (4.10)	18-35

Note: A significant or marginal difference from the old adult group is represented by (***) p<.001, (**) p<.01, (*) p<.05, (.) p<.1; A significant difference from the middle-aged group is represented by (+++) p<.001, (++) p<.01, (+) p<.05.

3. *AGE DIFFERENCES IN THE MPS-TOMQ TASK (TOM ITEMS)*

Regarding the ToM items of the task, the ANCOVA analyses showed significant effects of age groups for the reality ($F_{(2,302)}$=4.63, p=.01) and third-order false belief ($F_{(2,302)}$=4.54, p=.011) items, where the covariate, years of education, was not significant (reality: $F_{(1,302)}$=1.46, p=.23; 3rd order FB: $F_{(1,302)}$=.017, p=.90). In both these items, younger adults displayed a significantly better understanding and middle-aged adults a marginally greater

understanding than older adults. Furthermore, second-order false belief ($F_{(2,302)}$=4.77, p=.0091) items and the sequencing score ($F_{(2,302)}$=8.01, p<.001) showed age-related differences, although the years of education appeared to be significant ($F_{(1,302)}$=4.04, p=.045; $F_{(1,302)}$=13.22, p<.001, respectively). Younger adults had a marginally higher understanding of second-order false belief, whereas middle aged adults were significantly better than older adults. The opposite applies to the reality items were younger adults manifested a superior understanding and middle-aged adults a marginally higher understanding than older adults. Interestingly, the second-order belief and the first-order false belief items displayed an effect of years of education (2nd order belief: $F_{(1,302)}$=5.90, p=.016; 1st order FB: $F_{(1,302)}$=4.92, p=.027) but no effect of age groups (2nd order belief: $F_{(2,302)}$=1.53, p=.22; 1st order FB: $F_{(2,302)}$=.37, p=.69). Whereas the first-order belief items showed a marginal effect of age groups ($F_{(2,302)}$=2.53, p=.081) and no effect of years of education ($F_{(1,302)}$=.83, p=.36).

Predictably a main effect of age groups ($F_{(2,302)}$=9.91, p<.001) and years of education ($F_{(1,302)}$=10.49, p=.0013) arose on the total MPS-TOMQ score. Indeed, younger and middle-aged adults performed significantly better than older adults on the different items overall. Table S3 presents the descriptive statistics divided by age group.

Table S3 : Adjusted descriptive statistics for the ToM items of the MPS-TOMQ task divided by age group.

	**Younger adults**		**Middle-aged adults**		**Older adults**	
	Mean (SE)	Range	Mean (SE)	Range	Mean (SE)	Range
Sequencing (MPS)	27.8 (0.575)	16-36	27.52 (0.517)	17-36	24.7 $^{(**, ++)}$ (0.569)	11-36

1st order beliefs	2.59	2-4	2.86	0-4	2.61	0-4
	(0.1073)		(0.0964)		(0.1063)	
1st order FB	4.48	2-6	4.40	2-6	4.33	2-6
	(0.122)		(0.110)		(0.121)	
2nd order beliefs	3.53	2-4	3.70	1-4	3.61	0-4
	(0.0762)		(0.0685)		(0.0755)	
2nd order FB	5.08	2-6	5.16	1-6	4.55 (. , ++)	0-6
	(0.153)		(0.137)		(0.151)	
3rd order FB	3.20	0-4	2.97	0-4	2.55 (**,§)	0-4
	(0.147)		(0.132)		(0.145)	
reality	3.23	0-4	3.08	0-4	2.72 (*,§)	0-4
	(0.115)		(0.104)		(0.114)	
MPS-TOMQ	64.5	47-82	64.2	43-82	58.3 (***, +++)	28-81
	(1.089)		(0.979)		(1.078)	

A significant or marginal difference from the young adult group is represented by (***) *p*<.001, (**) *p*<.01, (*) *p*<.05, (.) *p*<.1;
A significant or marginal difference from the middle-aged group is represented by (+++) *p*<.001, (++) *p*<.01, (+) *p*<.05, (§) *p*<.1
